# Lightweight Super-Resolution with Self-Calibrated Convolution for Panoramic Videos

**DOI:** 10.3390/s23010392

**Published:** 2022-12-30

**Authors:** Fanjie Shang, Hongying Liu, Wanhao Ma, Yuanyuan Liu, Licheng Jiao, Fanhua Shang, Lijun Wang, Zhenyu Zhou

**Affiliations:** 1 Key Laboratory of Intelligent Perception and Image Understanding of Ministry of Education, School of Artificial Intelligence, Xidian University, Xi’an 710071, China; 2 The Medical College, Tianjin University, Tianjin 300072, China; 3 College of Intelligence and Computing, Tianjin University, Tianjin 300350, China; 4 Hangzhou Institute of Technology, Xidian University, Hangzhou 311231, China; 5 Hunan University of Science and Engineering, Yongzhou 425199, China

**Keywords:** panoramic videos, super-resolution, lightweight network, deformable convolution, self-calibration convolution

## Abstract

Panoramic videos are shot by an omnidirectional camera or a collection of cameras, and can display a view in every direction. They can provide viewers with an immersive feeling. The study of super-resolution of panoramic videos has attracted much attention, and many methods have been proposed, especially deep learning-based methods. However, due to complex architectures of all the methods, they always result in a large number of hyperparameters. To address this issue, we propose the first lightweight super-resolution method with self-calibrated convolution for panoramic videos. A new deformable convolution module is designed first, with self-calibration convolution, which can learn more accurate offset and enhance feature alignment. Moreover, we present a new residual dense block for feature reconstruction, which can significantly reduce the parameters while maintaining performance. The performance of the proposed method is compared to those of the state-of-the-art methods, and is verified on the MiG panoramic video dataset.

## 1. Introduction

Video super-resolution (VSR) is a classic problem in computer vision, and aims to recover high-resolution videos from low-resolution ones. VSR technology has been widely used in various areas for high-definition displays, such as network videos, digital TV, and surveillance drones. The panoramic video is one class of videos that are real 360-degree omnidirectional sequences, and its pixels are usually arranged in a spherical shape that can provide an immersive experience for users. The panoramic video is the product of a combination of multiple video technologies. Such a video allows the audience to see a wider field of view and a more realistic field of view experience. Because of its 3D stereoscopic characteristics compared with ordinary videos, it is widely used in entertainment, news, the military, and other fields.

In recent years, due to the emergence of deep learning, various methods for video super-resolution based on deep learning have been proposed. For instance, in SOFVSR [1], an optical flow reconstruction network is presented to infer high-resolution (HR) optical flow from coarse to fine, and the motion-compensated low resolution (LR) is input to a super-resolution network to generate the final super-resolved frames. TDAN [2] proposes a temporal deformable network, which utilizes the features of the reference frame and neighboring frames to dynamically predict the offset of the sampling convolutional kernel, and aligns it adaptively at the feature level. EDVR [3] proposes a pyramid, cascade, and deformable convolution (PCD) module. Unlike TDAN, this module performs alignment in a coarse-to-fine manner and can handle videos with large and complex motions. Moreover, EDVR presents the temporal and spatial attention fusion module, which utilizes temporal attention to concentrate on neighboring frames that are more similar to the reference frame, and uses spatial attention to assign weights to each position in each channel to more effective use of cross-channel and spatial information.

Although the methods mentioned above can achieve good performance for general videos, they may degrade for panoramic videos. Because panoramic videos usually have ultrahigh spatial resolution, they can provide viewers with a strong sense of immersion in the virtual environment. Moreover, the higher the resolution of the camera, the more realistic effect of the panoramic video is. The high resolution requires great hardware performance from camera equipment, and the cost will be largely increased. For this problem, Liu et al. [4] first explored deep learning for super-resolving panoramic videos. Although this method has gained a higher PSNR for panoramic videos, the number of parameters is still high. This issue considerably limits their real-world applications.

In order to balance the performance and the computational cost, we propose a novel lightweight super-resolution framework for panoramic videos. As is known, the alignment between video frames is significant for super-resolution. If more interframe information can be exploited for alignment, it is beneficial to the subsequent reconstruction. Thus, we present a new pooled, self-calibrated convolution (PSCC) for frame alignment, which significantly reduces the complexity of the deformable convolution and achieves accurate alignment in a gradual manner. Moreover, in the reconstruction operation, we design a new lightweight residual dense block to further reduce the complexity of the model. Our method achieves a balance between algorithm performance and complexity.

The main contributions of this work are listed as follows.

- We propose the first lightweight panoramic video super-resolution (LWPVSR) method for panoramic video super-resolution, which can achieve a good balance between performance and complexity. To the best of our knowledge, this is the first proposition of a lightweight panoramic VSR framework.
- Moreover, we present a new pooled, self-calibrated convolution for frame alignment. The self-calibrated convolution is introduced to make the learned offset more accurate in a progressive manner and reduce the complexity of the proposed network.
- Finally, we design a new significantly lighter residual dense block (LWRDB) for feature reconstruction, which achieves the purpose of reducing the complexity of the model while maintaining the performance of our method. Many experimental results verify the advantage of the proposed LWPVSR method against state-of-the-art methods.

The rest of this paper is organized as follows. Some related works on super-resolution of panoramic videos are introduced in Section 2. Section 3 describes the proposed lightweight super-resolution method in detail. In Section 4, we demonstrate the experimental results of our method. Finally, we show the conclusions and future work in Section 5.

## 2. Related Work

### 2.1. Super-Resolution Methods for Ordinary Videos

Most of video super-resolution methods (e.g., VESPCN [5], TDAN [2], SOFVSR20 [6], and EDVR [3]) have been proposed to address ordinary videos. They have improved the performance of restored high-resolution videos. For example, Yi et al. [7] proposed a general omniscient framework to leverage the LR framework and estimated hidden states from the past, present, and future frames. Benefiting from the global information feature of OVSR [7], the OVSR method refreshes the metrics on the Vid4 test set.

### 2.2. Super-Resolution Methods for Panoramic Videos

There are many image super-resolution methods such as [8,9,10,11,12,13]. For instance, ref. [11] can utilize the plenoptic geometry of the scene to perform alignment between consecutive frames in a video sequence and employ all visual information to generate high-resolution panoramic images. In [10], the spherical Fourier transform (SFT) was calculated based on the nonuniform sampling data on the sphere, which can transform low-resolution panoramic images with arbitrary rotation to reconstruct a high-resolution panoramic image. The joint alignment and super-resolution problem is converted into a least square minimization problem in the SFT domain. In [14], the authors introduced SRCNN [15], which is the earlier work to use deep learning for super-resolution of panoramic images. It fine tuned the SRCNN by optimizing input size and using the panoramic training set to adapt the fine-tuned method to the features of the high-resolution panoramic images. Based on the existing viewport-based panoramic image transmission system, ref. [16] proposed a framework that used the high-resolution content of the viewport to improve the quality of the surrounding low-resolution areas. The adaptive initial viewport of each image was predicted in view of contextual similarity of the sphere, so as to provide more useful information for low-resolution regions.

Only a few works involve the super-resolution of panoramic videos. As we have mentioned in Section 1, Liu et al. [4] designed a single frame and multiframe joint network for the super-resolution of panoramic videos, which explored both the spatial information and the temporal information. In addition, deformable convolutions are employed to eliminate the motion difference between feature maps of the target frame and its neighboring frames. Although it achieves sound results, the network contains a large number of parameters, resulting in low computational efficiency, which is not unfavorable for the promotion of practical applications. Therefore, this paper will propose the first lightweight video super-resolution method for panoramic videos.

## 3. The Proposed Lightweight Architecture for Panoramic Video Super-Resolution

In this section, we propose the first lightweight panoramic video super-resolution (LWPVSR) method. The proposed LWPVSR method mainly consists of the four main modules: the feature extraction module, the feature alignment module, the reconstruction module, and the dual network module, as shown below.

### 3.1. Our Network Architecture

**Figure 1:**
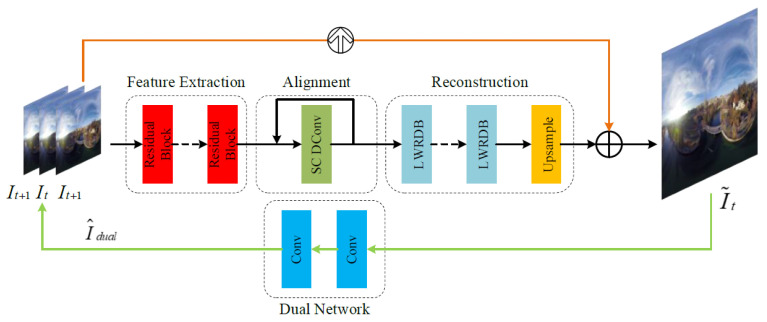
The network architecture of the proposed LWPVSR method. Our LWPVSR method mainly consists of the four modules: the feature extraction module, the feature alignment module, the reconstruction module, and the dual network module.

As shown in Figure 1, the network structure of our method mainly consists of three main parts, which are the feature extraction module, the feature alignment module, and the reconstruction module. The backbone network learns the residual images of the video frames, then sums them with the direct upsampling results of the target frames to obtain the final super-resolution results. In addition, super-resolution is an ill-posed problem—that is, mapping a low-resolution video to high-resolution is a one-to-many problem. In order to reduce the solution space of the super-resolution, we introduce a dual mechanism to the backbone network, and it learns a dual regression mapping, which can increase the constraints on LR videos—that is, the duality mechanism acts as a subsupervised network to enhance the performance of SR. The whole super-resolution process of the proposed method is expressed as follows,

(1) $${\tilde{I}}_{t}=H_{LWPVSR}\left({\hat{I}}_{t-N:t+N}\right),$$

where $H_{LWPVSR}\left(\cdot \right)$ denotes the proposed algorithm network, *N* is the temporal radius (e.g., N=3), and ${\tilde{I}}_{t}$ and ${\hat{I}}_{t}$ are the super-resolution result of the target frame and the low resolution of the target frame, respectively.

### 3.2. The Feature Extraction Module

The feature extraction module is responsible for extracting the features of the input video frames to prepare for subsequent feature alignment. In the proposed method, the feature extraction module is composed of several residual blocks, which mainly consist of two convolutional layers. The residual block is more conducive to training. Therefore, the number of parameters is very small, and it maintains network performance in combination with other modules. The process of the proposed feature extraction module can be formulated as follows:

(2) $$F=H_{FE}\left({\hat{I}}_{t-N:t+N}\right).$$

Note that $H_{FE}\left(\cdot \right)$ denotes the feature extraction operation, and *F* denotes the extracted features.

### 3.3. The Proposed Feature Alignment Module

**Figure 2:**
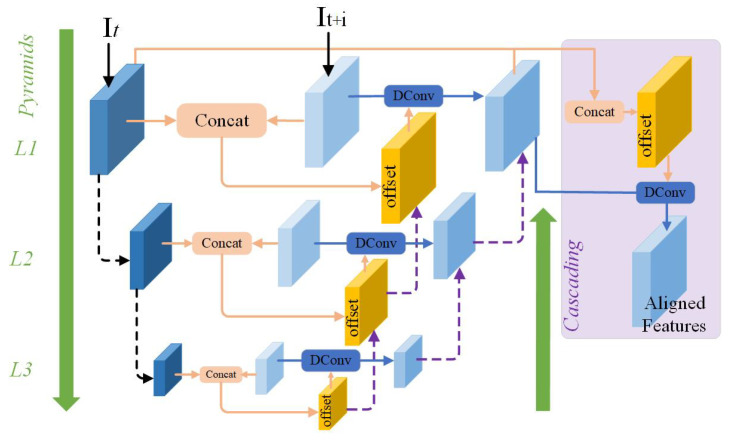
The structure of the PCD module in EDVR [3].

**Figure 3:**
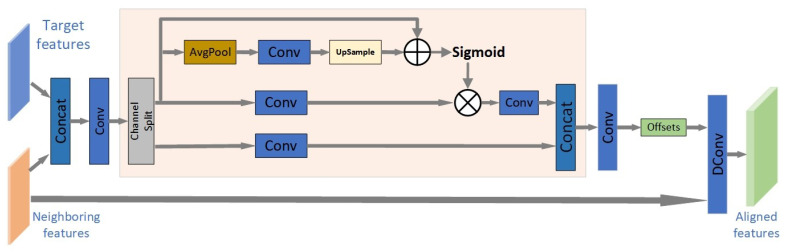
Our proposed pooled self-calibrated convolution (PSCC) module for feature alignment.

In this subsection, we propose a new module for feature alignment between frames based on deformable convolution. As is known, the effectiveness of deformable convolution in video super-resolution has been witnessed and confirmed in EDVR [3]. In EDVR, the deformable convolution was integrated into a pyramid, cascading, and deformable convolution (PCD) module in EDVR, as shown in Figure 2. In fact, PCD has a pyramid-like structure. The top layer is a lower-resolution feature map, and the bottom layer is for the reference frame and neighboring frames. Different layers represent the feature information of different frequencies. PCD first aligns the reference feature map with the smallest resolution to form a rough alignment, and then transfers the offset and aligned feature map to a layer with a larger resolution, so that the offset and continuously aligned feature map are passed to the bottom layer every time. Thus, an implicit motion compensation from coarse to fine is formed from top to bottom. However, PCD introduced a large number of parameters and computational cost. In order to reduce the parameters, here we design a new pooled, self-calibrated convolution (PSCC) to replace the pyramid cascading structure and maintain the multi-scale learning capability, as shown in Figure 3.

Inspired by the self-calibrated convolution in [17], our PSCC module employs a pooling for downsampling to expand the receptive field, so as to learn more contextual information without increasing the network complexity. Specifically, after the target features and the neighboring features are merged, a convolution operation is performed, and then through channel splitting, the channel is divided into two, one channel only performs a simple convolution. In the other channel, we utilize a new upsample operation to make the learned features match with the scale. The learned features via the Sigmoid activation are again multiplied with the features through channel splitting. Finally, the features from the two channels are concatenated for output. Our PSCC module is embedded in the deformable convolution to learn the information about the neighbors of the reference frame, rather than the global information, so as to avoid the pollution information of other irrelevant frames and achieve more accurate frame alignment. From the perspective of the structures of PCD and PSCC, PCD uses multiple deformable convolutional networks (DCNs) and convolutional networks to cascade and form a pyramid structure, and each DCN is based on a feature map of different levels. Although our PSCC only adopts one deformable convolutional network, and combines it with self-correcting convolution, which not only reduces the number of parameters, but also learns more accurate offsets to achieve better alignment. This will be verified by the subsequent experimental results. Compared with the PCD module with 1.38 M parameters in EDVR, the number of the parameters of our PSCC module is only 0.04 M.

The process of the feature alignment in our LWPVSR method is expressed as follows,

(3) $$F_{t\pm i}^{a}=H_{Alignment}\left(F_{t},F_{t\pm i}\right),$$

where $H_{Alignment}\left(\cdot \right)$ denotes our alignment operation and $F_{t}$ and $F_{t\pm i}$ denote the features of the target frame and the nearest neighboring frame, respectively. $F_{t\pm i}^{a}$ denotes the aligned features of each frame. Here, we use $F^{a}$ to represent the result of all the aligned frames.

### 3.4. Our Reconstruction Module

**Figure 4:**
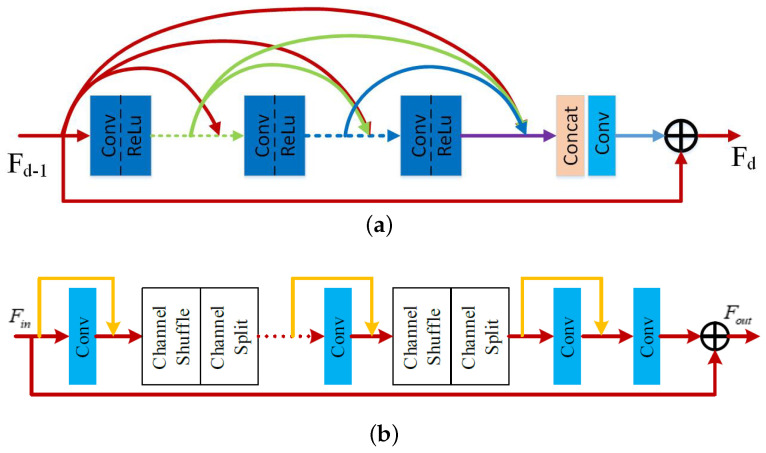
Comparison of the structures of the residual dense block (RDB) used in [19,20] and our lightweight RDB. (**a**) Existing RDB [19,20]. (**b**) Our lightweight RDB.

In the proposed reconstruction module, inspired by the residual dense blocks (RDB) in [18], as shown in Figure 4a, a lightweight residual dense block (LWRDB) is designed for feature transformation and restoration. Its detailed structure is shown in Figure 4b.

First, the input features $F_{in}$ through a layer of convolution output the corresponding feature maps. The channel is then shuffled by a channel shuffle operation, followed by a channel split operation which divides the number of channels into two proportionally. One part is fed into the convolution, and the other is connected to the output of the convolution in a jump connection. The channel shuffle and channel split operations are executed again, and so on. Finally, the number of channels is reduced through a 1 × 1 convolution, and the output is added to the initial input of the module to obtain the final result $F_{out}$ of the module. The process is given by

(4) $$F_{out}=H_{LWRDB}\left(F_{in}\right),$$

where $H_{LWRDB}\left(\cdot \right)$ denotes the operation of the LWRDB module. It is noted that in our design, the introduction of the channel shuffle [19] and the channel split [20] is important compared with that of Figure 4a. The purpose of the channel shuffle operation is to break up the output of the previous layer of convolution in channel dimension, and the purpose of the channel split operation is to split the channel into two according to a presetting ratio, as shown in Figure 4b. The purpose of combining these two operations is to reduce the number of parameters while still making full use of all levels of features like residual dense blocks, so that the number of parameters can be reduced. Meanwhile, it maintains high performance. It should be noted that we have verified in our experiment that the number of parameters of the RDB module is 1.08 M, while that of ours is only 0.81 M. Obviously, the number of parameters of our proposed LWRDB is smaller. In fact, our lightweight residual block in the reconstruction module introduces both channel shuffle and channel split operations. The channel shuffle can strengthen the exchange of information between channels, and channel split can reduce the number of parameters. Compared with the reconstruction module in Figure 4a, our method has fewer parameters and can maintain the performance of the network.

### 3.5. Our Dual Network Module and Loss Function

In our proposed method, the final super-resolution result is obtained by adding the output of the reconstruction module to the result of the upsampled target frame. It is expressed as follows,

(5) $${\tilde{I}}_{t}=H_{reconst}\left(F^{a}\right)+{\hat{I}}_{t}↑,$$

where $H_{reconst}\left(\cdot \right)$ denotes the mapping function of reconstruction module. Here ↑ denotes upsampling, and ${\tilde{I}}_{t}$ denotes the super-resolution result of the target frame.

In order to show the important content in the equatorial region in the panorama video, we introduce a weighted mean square error (WMSE) loss, which is defined as follows,

(6) $$\frac{1}{\sum_{i=0}^{M-1}\sum_{j=0}^{N-1}\omega \left(i,j\right)}\sum_{i=0}^{M-1}\sum_{j=0}^{N-1}\omega \left(i,j\right)\cdot {\left({\tilde{I}}_{t}\left(i,j\right)-I_{t}\left(i,j\right)\right)}^{2},$$

where *M* and *N* denote the width and height of one frame, respectively, (i,j) represents the coordinate position of each pixel in a frame, and ω(i,j) is the weight at the corresponding pixel position, which is allocated according to the pixel position and is given by

(7) $$\omega \left(i,j\right)=\cos\frac{(j+0.5-\frac{N}{2})\pi }{N},$$

where $i=i_{0},i_{0}+1,\cdots ,i_{0}+wd-1$ and $j=j_{0},j_{0}+1,\cdots ,j_{0}+h-1$. Here, $\left(i_{0},j_{0}\right)$ represents the upper left corner of the patch, wd is the width of the patch, and *h* is the height of the patch.

**Figure 5:**
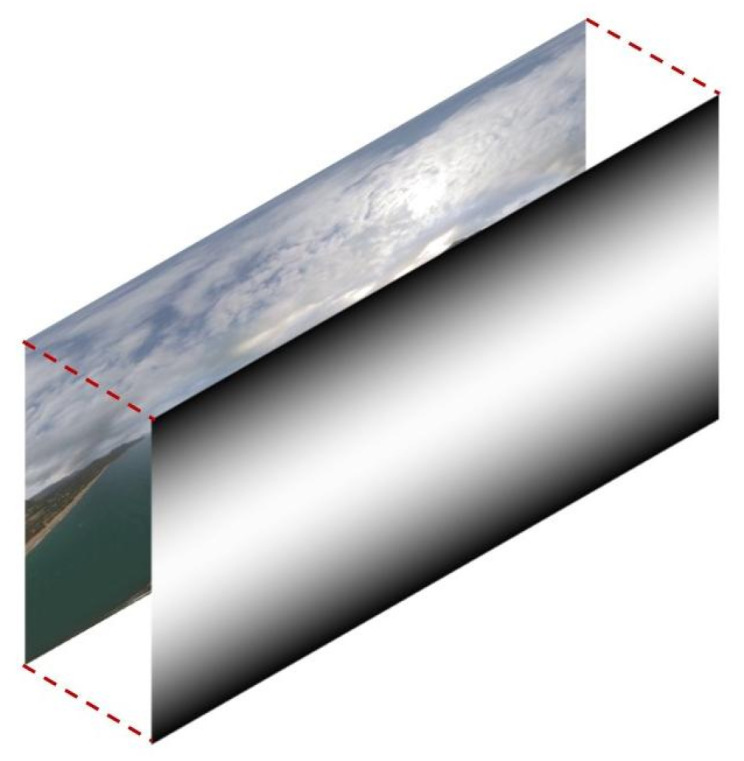
The weight diagram of the loss function.

In order to explain the weight change in each frame more intuitively, we show it visually in Figure 5. The black and white color represent the distribution of weights. The lighter the color, the greater the weight is, and the darker the color, the smaller the weight is. That is, the weights gradually decrease from the equator to the two polar regions. The weights are assigned on the whole frame during data processing.

The loss function in our architecture is composed of two parts. One is from the main branch—$L_{primary}$ (i.e., input, feature extraction, alignment, reconstruction, and output)—and the other is from the dual subnetwork: $L_{dual}$. The overall loss function of the proposed method is formulated as follows,

(8) $$L_{total}=L_{primary}+\lambda L_{dual},$$

where $L_{primary}$ and $L_{dual}$ are both calculated by Equation (6). The parameter λ is a balance factor between $L_{primary}$ and $L_{dual}$.

It is noted that compared with ordinary videos, the information of panoramic video is distributed on a sphere instead of a plane. The panoramic video, which is essentially a spherical video, cannot directly use the storage structure and encoding algorithm designed for ordinary videos. The current mainstream solution is to use the mapping relationship to project the spherical video onto the plane and compress the obtained plane video, and the equirectangular projection (ERP) is widely used. In this case, the important content is usually displayed in the equatorial region, and the less content is at the poles. Moreover, because the information of the panoramic video is distributed in a spherical shape, the features in the same dimensionality are more uneven, and the video is more prone to be distorted. Addressing the particularity that more content distributed at the equator and less content at the poles, we used the weighted loss function, as shown in Equation (6). It aims to increase the weight of the equatorial region and reduce the weight of the polar region. Addressing the features distributed on the same dimension are more uneven or the offset is too large, we think that using deformable convolution is not sufficient to solve this issue. Therefore, we propose to adopt self-correcting convolution combined with deformable convolution—that is, our PSCC module to learn these offset features. It is more conducive to achieving better alignment results.

## 4. Experimental Results

In this section, we compare the proposed LWPVSR method with eight state-of-the-art super-resolution algorithms for panoramic video super-resolution tasks.

### 4.1. Datasets

The MiG dataset [4] is utilized for evaluating the performance of super-resolution of the proposed LWPVSR method. The data set has 200 videos for training and eight videos for test. We adopt the bicubic interpolation algorithm to 2× downsample each video frame as the ground truth (GT). Then, we further perform 4× downsampling on GT to obtain the corresponding LR video. Moreover, in order to demonstrate the superiority of the proposed LWPVSR method, we also collected another video sequence from the Internet, named Clip_009, and adopted it for performance evaluation.

The peak signal-to-noise ratio (PSNR) and structural similarity (SSIM) are usually used as indicators to measure the performance of all the video super-resolution algorithms. In order to make a fair comparison, similar to other works, all indices are calculated on the Y channel for all the algorithms. Different from ordinary videos, we also use the two video quality metrics (i.e., WS-PSNR and WS-SSIM) in [4] to measure the performance of all the methods.

### 4.2. Training Setting

We implemented all the models in the PyTorch framework and used two NVIDIA Titan XP GPUs for training. The training schemes and parameters of other methods are listed below.

(1) SR360 [8]: The batch size is set to 16. The weights of all the layers were initialized randomly and the network was trained from the scratch. The network used the Adam solver with a learning rate, $1\times 10^{-4}$.
(2) VSRnet [21]: The batch size is 240, a learning rate of is $1\times 10^{-4}$ used for the first two layers, $1\times 10^{-5}$ for the last layer and a weight decay rate of 0.0005 are set as in [21].
(3) FRVSR [22]: The Adam is an optimizer. The learning rate is fixed at $1\times 10^{-4}$. Each sample in the batch is a set of 10 consecutive video frames, i.e., 40 video frames are passed through the networks in each iteration.
(4) VESPVN [5]: The initial batch size is 1. Every 10 epochs the batch size is doubled until it reaches a maximum size of 128. The optimizer is Adam with a learning rate, $1\times 10^{-4}$.
(5) TDAN [2]: The batch size is set to 64. The Adam is the optimizer. The learning rate is initialized to $1\times 10^{-4}$ for all layers and decreases half for every 100 epochs.
(6) SOFVSR [6]: The batch size is 32. The optimizer is Adam. The initial learning rate is $1\times 10^{-3}$ and divided by 10 after every 80 K iterations.
(7) EDVR [3]: The batch size is set to 32. The learning rate is initialized to $4\times 10^{-4}$, and initializes deeper networks by parameters from shallower ones for faster convergence.
(8) OVSR [7]: The batch size is 16. The optimizer is Adam. The initial learning rate is $1\times 10^{-3}$ and decays linearly to $1\times 10^{-4}$ after 120 K iterations, which keeps the same until 200 K iterations. Then the learning rate is further decayed to $5\times 10^{-5}$ and $1\times 10^{-5}$ until convergence.

In our method, the feature extraction module is composed of three residual blocks, each residual block consists of two layers of convolution, and the number of channels is set to 64. The reconstruction module includes five LWRDB blocks, each block is composed of six convolutional layers, and the number of channels is 64. In our experiment, we convert the video frames from the RGB space to the YCbCr space and then use the Y channel as the input to our network. Unless stated otherwise, the network takes three consecutive video frames as inputs. The input patch size is 64 × 64, and the batch size is set to 32. Moreover, we also employ data enhancement techniques as in other methods, including reflection, random cropping, and rotation. Furthermore, we defined the ratio for channel split by experience. If the ratio is larger than 0.5, it means that more features do not participate in the subsequent calculations but are directly cascaded to the subsequent feature maps. Then the following convolutional layers will be meaningless. If the ratio is smaller than 0.5, the model parameters will increase and it results in a higher computational cost. Therefore, the ratio equaling to 0.5 is a balanced choice. During training, we optimize the network by using the Adam optimizer with $\beta_{1}=$ 0.9 and $\beta_{2}=$ 0.999. The initial learning rate is set to $2\times 10^{-4}$, and then is reduced by half after every 20 epochs. In our loss function, through experiments and experiences, the value of the parameter λ is set to 0.1. And the performance of each method has been optimized with its hyperparameter tuning to show their best results in our experiments.

### 4.3. Quantitative Comparison

We also implemented nine other state-of-the-art VSR algorithms for performance comparison. They include bicubic, SR360 [8], VSRnet [21], VESPCN [5], FRVSR [22], TDAN [2], SOFVSR20 [6], EDVR [3], and OVSR [7]. The quantitative results including PSNR/WS-PSNR, SSIM/WS-SSIM, inference time, and floating point operations per second (FLOPs) of all the methods on representative video clips are shown in Table 1 and Table 2, respectively.

It can be seen that our LWPVSR method obtains the highest PSNR and SSIM results, and the amount of its parameters is relatively small. Our LWPVSR method performs much better than EDVR in terms of PSNR/WS-PSNR and SSIM/WS-SSIM, and the the former has significantly fewer parameters than the latter (i.e., 2.30 M vs. 20.60 M). That is, LWPVSR is nearly 1/10 size of EDVR. It is because the proposed PSCC module in our LWPVSR plays an important role, and decreases the PCD module in EDVR by man parameters but maintains the performance. In addition, compared with FRVSR, our model parameters are 2.7 M smaller, and the PSNR of model is 1.65 dB higher than FRVSR. SR360, VSRnet, VESPCN, TDAN, and SOFVSR20 are relatively lightweight video super-resolution architectures, with model parameters below 2.0 M. However, the performance of all of them is significantly lower than that of the proposed method. Moreover, the PSNR and WS-PSNR results of all the methods on other video clips are shown in Table 3 and Table 4. We can see that the results of our LWPVSR method are much better than those of the state-of-the-art methods. All the experimental results show that our LWPVSR method can achieve a good balance between the model complexity and performance.

**Figure 6:**
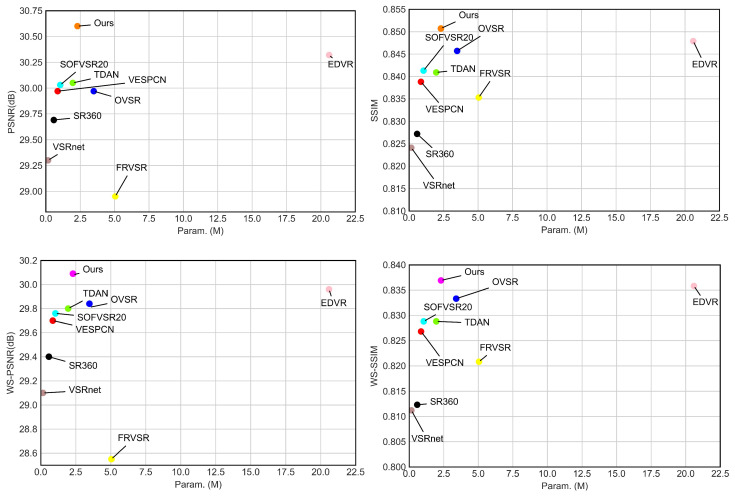
Comparison of all the methods in terms of performance and number of parameters. Note that the *y*-axis represents different performance metrics (including PSNR, SSIM, WS-PSNR, and WS-SSIM), and the *x*-axis corresponds to the number of parameters in different methods.

In order to demonstrate the relation between performance and parameters more clearly, the visualized diagram is also shown in Figure 6. It can be seen that our method attains a higher performance at the cost of lower numbers of parameters.

### 4.4. Qualitative Comparison

**Figure 7:**
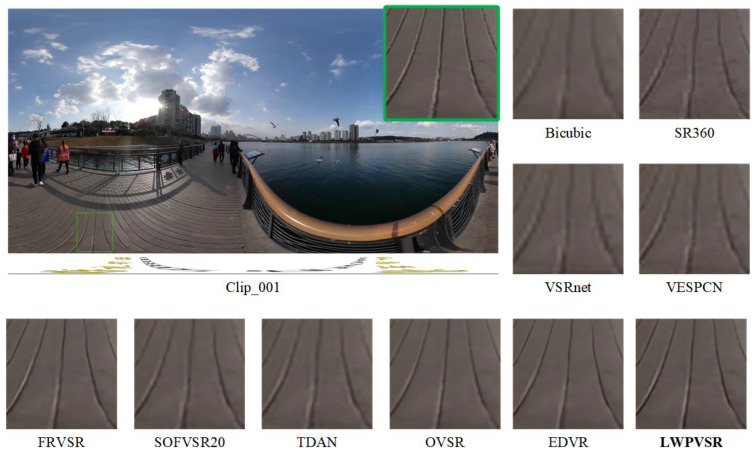
The results of all the algorithms performing 4× super-resolution on Clip_001 of the MiG test set.

**Figure 8:**
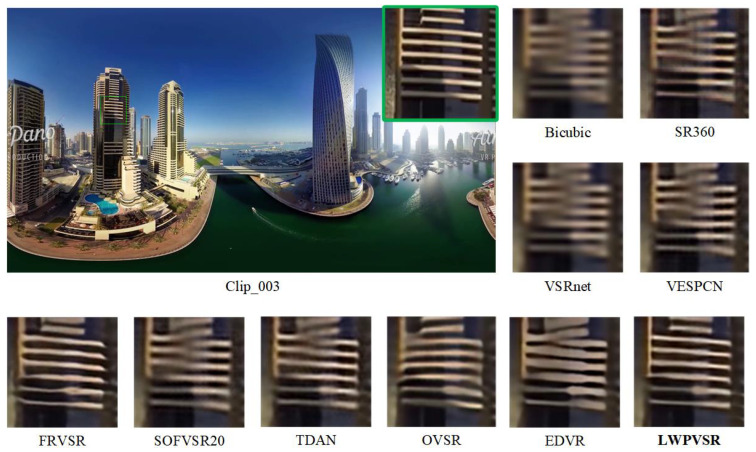
The results of all the algorithms performing 4× super-resolution on Clip_003 of the MiG test set.

**Figure 9:**
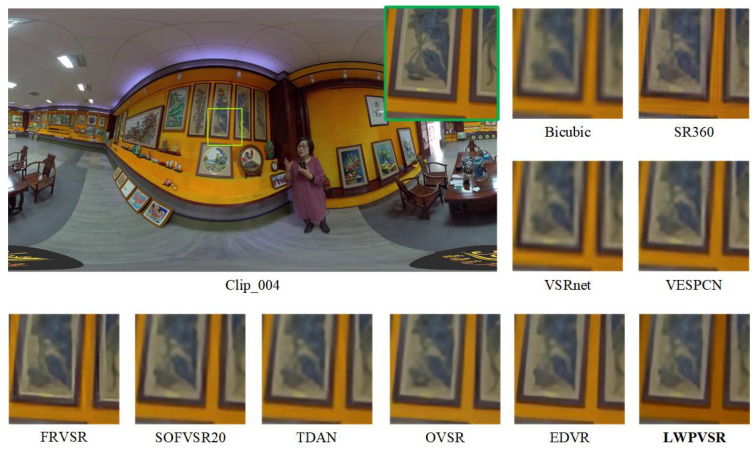
The results of all the algorithms performing 4× super-resolution on Clip_004 of the MiG test set.

**Figure 10:**
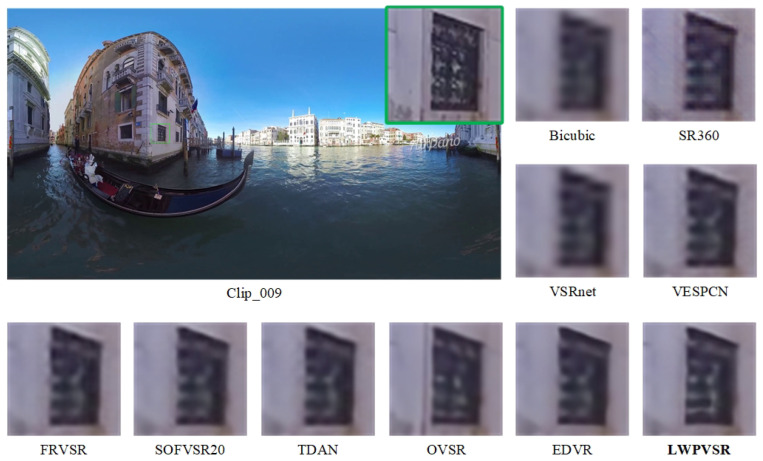
The results of all the algorithms performing 4× super-resolution on Clip_009 of the MiG test set.

In this subsection, we qualitatively compare our method with the other methods on video sequences Clip_001, Clip_003, Clip_004 and Clip_009, as shown in Figure 7, Figure 8, Figure 9 and Figure 10, respectively.

It can be seen that our LWPVSR method has achieved much better performance than other methods, including EDVR with 20.60 M parameters, and they have superior visual results in all these figures. For example, in Figure 7, the image recovered by our LWPVSR method seems more real, which is closer to the original high-resolution image. However, the images recovered by other methods, such as TDAN, FRVSR, and SOFVSR20, are blurry. Similar results can also be observed from Figure 8, Figure 9 and Figure 10. In general, compared with other methods, our LWPVSR method achieves a better balance between the model complexity and algorithm performance, resulting in less distortion and more reliable results in the panoramic video super-resolution task.

### 4.5. Ablation Studies

In this subsection, we analyze the contribution of each module in our network, mainly including PSCC and LWRDB, as shown in Table 5. The baseline is our architecture, as shown in Figure 1. The PSNR and SSIM results are 30.60 dB and 0.8507, respectively. When the architecture is without the PSCC module, the PSNR drops by 0.30 dB, and the number of parameters decreases 0.04 M. The performance drops by 0.92 dB when the baseline is without the LWRDB module. Moreover, without PSCC and LWRDB, the PSNR result decreases by 0.96 dB. All the results also verify the importance of the proposed modules, including PSCC and LWRDB for the proposed method.

## 5. Conclusions and Future Work

In this paper, a lightweight and efficient panoramic video super-resolution method was designed from the perspective of lightweight networks. This method adopts deformable convolution to align the nearest neighbor features with the target feature, in order to further enhance the alignment effect step. In particular, we introduced self-calibrated convolution to gradually implement the alignment operation in a recursive manner. Moreover, we also proposed a lighter and more efficient LWRDB module based on the RDB module. Various experimental results verified the effectiveness of the proposed method. Compared with mainstream video super-resolution algorithms, our proposed method achieves a better balance between performance and algorithm complexity.

In the future, we will design more effective strategies, such as the attention strategy [23] for the lightweight architecture to further enhance the performance while maintaining its cost.

## Figures and Tables

**Table 1:** Comparison of all the methods in terms of PSNR (top) and SSIM (bottom).

	Bicubic	SR360 [8]	VSRnet [21]	FRVSR [22]	VESPCN [5]	TDAN [2]	SOFVSR20 [6]	EDVR [3]	OVSR [7]	LWPVSR
Clip_005	26.38	26.58	26.59	25.36	26.71	26.73	26.72	26.73	26.69	**26.81**
	0.6868	0.7101	0.7075	0.7095	0.7203	0.7213	0.7203	0.7217	0.7207	**0.7251**
Clip_006	30.09	30.69	30.39	29.70	31.04	31.11	31.16	31.48	29.72	**31.58**
	0.8494	0.8580	0.8573	0.8700	0.8723	0.8740	0.8775	0.8685	0.8902	**0.8902**
Clip_007	27.65	29.29	28.10	28.90	29.50	29.61	29.54	30.18	29.31	**30.99**
	0.8119	0.8406	0.8245	0.8458	0.8490	0.8534	0.8527	0.8630	0.8622	**0.8700**
Clip_008	31.88	32.22	32.15	31.83	32.63	32.74	32.70	32.91	32.81	**33.02**
	0.9005	0.9001	0.9069	0.9162	0.9134	0.9150	0.9147	0.9186	0.9183	**0.9183**
Average	29.00	29.69	29.30	28.95	29.97	30.05	30.03	30.32	29.97	**30.60**
	0.8121	0.8272	0.8241	0.8353	0.8388	0.8409	0.8413	0.8479	0.8457	**0.8507**
Params. (M)	-	0.58	0.16	5.05	0.86	1.96	1.05	20.60	3.48	2.30
Time (ms)	-	64.30	2.52	71.57	122.59	16.11	76.86	670.80	69.55	92.31
FLOPs (T)	-	0.457	0.018	0.348	0.007	0.558	0.135	0.954	0.201	0.204

**Table 2:** Comparison of all the methods in terms of WS-PSNR (top) and WS-SSIM (bottom).

	Bicubic	SR360 [8]	VSRnet [21]	FRVSR [22]	VESPCN [5]	TDAN [2]	SOFVSR20 [6]	EDVR [3]	OVSR [7]	LWPVSR
Clip_005	26.39	26.62	26.60	25.37	26.75	26.78	26.77	26.80	26.73	**26.84**
	0.6888	0.7131	0.7118	0.7257	0.7263	0.7267	0.7257	0.7293	0.7260	**0.7298**
Clip_006	28.64	29.37	28.94	28.27	29.63	29.71	29.74	30.04	29.72	**30.15**
	0.8274	0.8422	0.8386	0.8574	0.8569	0.8594	0.8622	0.8744	0.8685	**0.8759**
Clip_007	29.75	30.76	30.15	30.23	31.24	31.42	31.29	31.57	31.55	**31.86**
	0.8009	0.8214	0.8165	0.8374	0.8379	0.8406	0.8392	0.8464	0.8465	**0.8482**
Clip_008	30.46	30.85	30.72	30.34	31.19	31.28	31.24	31.43	31.34	**31.52**
	0.8685	0.8726	0.8779	0.8869	0.8854	0.8885	0.8880	0.8929	0.8924	**0.8938**
Average	28.81	29.40	29.10	28.55	29.70	29.80	29.76	29.96	29.84	**30.09**
	0.7964	0.8123	0.8112	0.8268	0.8266	0.8288	0.8288	0.8358	0.8333	**0.8369**
Params. (M)	-	0.58	0.16	5.05	0.86	1.96	1.05	20.60	3.48	2.30
Time (ms)	-	64.30	2.52	71.57	122.59	16.11	76.86	670.80	69.55	92.31
FLOPs (T)	-	0.457	0.018	0.348	0.007	0.558	0.135	0.954	0.201	0.204

**Table 3:** Comparison of all the methods in terms of PSNR (top) and SSIM (bottom) on other video clips.

	Bicubic	SR360 [8]	VSRnet [21]	FRVSR [22]	VESPCN [5]	TDAN [2]	SOFVSR20 [6]	EDVR [3]	OVSR [7]	LWPVSR
Clip_001	27.57	29.06	27.75	28.80	28.56	29.20	29.08	29.44	29.25	**29.68**
	0.8659	0.8833	0.8742	0.8920	0.8909	0.8965	0.9004	0.9108	0.9122	**0.9176**
Clip_002	26.06	27.26	26.54	27.42	27.20	27.43	27.39	27.58	27.87	**27.75**
	0.7426	0.7866	0.7650	0.8045	0.7976	0.8052	0.8073	0.8138	0.8378	**0.8231**
Clip_003	25.68	26.45	25.95	26.39	26.52	26.63	26.55	26.66	26.57	**26.82**
	0.8240	0.8495	0.8359	0.8551	0.8568	0.8623	0.8607	0.8663	0.8737	**0.8700**
Clip_004	30.61	31.46	31.08	32.25	32.17	32.44	32.46	33.03	32.72	**33.66**
	0.8889	0.8931	0.8983	0.9220	0.9196	0.9257	0.9280	0.9379	0.9404	**0.9412**
Clip_009	26.03	27.23	26.50	27.40	27.16	27.40	27.36	27.56	27.79	**29.41**
	0.7515	0.7957	0.7717	0.8123	0.8044	0.8131	0.8136	0.8224	0.8637	**0.8801**
Average	27.19	28.29	27.56	28.45	28.32	28.62	28.57	28.85	28.84	**29.46**
	0.8146	0.8416	0.8290	0.8572	0.8539	0.8606	0.8620	0.8702	0.8856	**0.8864**
Params. (M)	-	0.58	0.16	5.05	0.86	1.96	1.05	20.60	3.48	2.30
Time (ms)	-	64.30	2.52	71.57	122.59	16.11	76.86	670.80	69.55	92.31
FLOPs (T)	-	0.457	0.018	0.348	0.007	0.558	0.135	0.954	0.201	0.204

**Table 4:** Comparison of all the methods in terms of WS-PSNR and WS-SSIM on other video clips.

	Bicubic	SR360 [8]	VSRnet [21]	FRVSR [22]	VESPCN [5]	TDAN [2]	SOFVSR20 [6]	EDVR [3]	OVSR [7]	LWPVSR
Clip_001	29.84	30.86	30.19	30.95	31.12	31.35	31.35	31.89	31.67	**32.04**
	0.9630	0.8771	0.8731	0.8909	0.8901	0.8948	0.8969	0.9082	0.9053	**0.9071**
Clip_002	25.81	27.02	26.27	27.12	26.89	27.12	27.03	27.32	27.59	**27.45**
	0.7416	0.7792	0.7626	0.7975	0.7916	0.7978	0.8003	0.8082	0.8226	**0.8112**
Clip_003	24.49	25.17	24.75	25.12	25.23	25.33	25.26	25.37	25.26	**25.48**
	0.7807	0.8134	0.7972	0.8197	0.8212	0.8275	0.8252	0.8339	0.8308	**0.8312**
Clip_004	29.88	30.87	30.39	31.66	31.59	31.91	31.90	32.45	32.18	**32.89**
	0.8666	0.8802	0.8796	0.9077	0.9053	0.9122	0.9143	0.9249	0.9242	**0.9263**
Clip_009	25.78	26.99	26.24	27.09	26.87	27.09	27.03	27.29	27.98	**28.11**
	0.7448	0.7815	0.7617	0.7971	0.7907	0.7972	0.7993	0.8076	0.8312	**0.8387**
Average	27.16	28.18	27.57	28.39	28.34	28.56	28.51	28.86	28.94	**29.20**
	0.7993	0.8263	0.8148	0.8426	0.8398	0.8459	0.8472	0.8566	0.8628	**0.8629**
Params. (M)	-	0.58	0.16	5.05	0.86	1.96	1.05	20.60	3.48	2.30
Time (ms)	-	64.30	2.52	71.57	122.59	16.11	76.86	670.80	69.55	92.31
FLOPs (T)	-	0.457	0.018	0.348	0.007	0.558	0.135	0.954	0.201	0.204

**Table 5:** Ablation studies for each module in the proposed LWRDB network.

	PSNR	SSIM	Parameters (M)
Ours	30.60	0.8507	2.30
Ours w/o PSCC	30.30	0.8471	2.26
Ours w/o LWRDB	29.68	0.8290	1.49
Ours w/o PSCC and LWRDB	29.64	0.8285	1.44
